# *In vivo* assembly of DNA-fragments in the moss, *Physcomitrella patens*

**DOI:** 10.1038/srep25030

**Published:** 2016-04-29

**Authors:** Brian Christopher King, Konstantinos Vavitsas, Nur Kusaira Binti Khairul Ikram, Josephine Schrøder, Lars B. Scharff, Björn Hamberger, Poul Erik Jensen, Henrik Toft Simonsen

**Affiliations:** 1 Copenhagen Plant Science Centre, VILLUM Research Center for Plant Plasticity, Center for Synthetic Biology “bioSYNergy”, Department of Plant and Environmental Sciences, University of Copenhagen, Thorvaldsensvej 40, DK-1871 Frederiksberg C, Denmark; 2 Institute of Biological Sciences, Faculty of Science, University of Malaya, Kuala Lumpur, Malaysia; 3 Department of Systems Biology, Technical University of Denmark, Søltofts Plads, 2800 Kgs. Lyngby, Denmark

## Abstract

Direct assembly of multiple linear DNA fragments via homologous recombination, a phenomenon known as *in vivo* assembly or transformation associated recombination, is used in biotechnology to assemble DNA constructs ranging in size from a few kilobases to full synthetic microbial genomes. It has also enabled the complete replacement of eukaryotic chromosomes with heterologous DNA. The moss *Physcomitrella patens*, a non-vascular and spore producing land plant (Bryophyte), has a well-established capacity for homologous recombination. Here, we demonstrate the *in vivo* assembly of multiple DNA fragments in *P. patens* with three examples of effective genome editing: we (i) efficiently deleted a genomic locus for diterpenoid metabolism yielding a biosynthetic knockout, (ii) introduced a salt inducible promoter, and (iii) re-routed endogenous metabolism into the formation of amorphadiene, a precursor of high-value therapeutics. These proof-of-principle experiments pave the way for more complex and increasingly flexible approaches for large-scale metabolic engineering in plant biotechnology.

*P. patens* has been used for decades to study plant metabolic engineering^1^. The dominant phase of the life cycle is haploid, so backcrossing to establish homozygous transgenic lines is not necessary^1^. In addition, the genome has been fully sequenced^2^. The unique capacity of *P. patens* among plants for efficient homologous recombination has established *P. patens* as a model organism, especially for studies concerning the elucidation of gene function and the colonization of terrestrial ecosystems by plants^2^,^3^,^4^,^5^. Moreover, *P. patens* is used in biotechnology as a heterologous expression host for recombinant therapeutic proteins and small natural products of commercial value^1^,^6^,^7^,^8^,^9^. Plant specific glycosylation patterns can be immunogenic for injected therapeutics, however *P. patens* has been be precisely engineered to alter the glycosylation pattern of the recombinant proteins making them more human-like, thus providing an advantage to *P. patens*-derived therapeutics^1^,^6^. *P. patens* can grow in liquid cultures and large bioreactors in its filamentous haploid vegetative state that assures homogeneity and reproducibility of production^10^. Finally, *P. patens*, as a photosynthetic organism, has a high potential as biotechnological host, especially in cases when: the availability of light-dependent reduction equivalents is critical; the presence of subcellular compartments (such as plastids, mitochondria, and vacuoles) can be exploited for creating tailored microenvironments; and as an expression system for plant biosynthetic pathways^11^,^12^,^13^. Further development of genetic engineering methodologies could help to increase the power and potential of the platform.

Polyethylene glycol (PEG) mediated transformation of *P. patens* protoplasts with a single linearized plasmid is a standard method of introducing heterologous DNA into cells of *P. patens*^14^,^15^. *P. patens* also permits targeting of multiple insertion sites in one single transformation event^16^, and the homologous recombination efficiency is comparable to the yeast *Saccharomyces cerevisiae*^17^. Targeted integration in the nuclear genome typically utilizes homologous sequences between the genome and inserted DNA of 500 bases or longer^14^.However, it has been previously demonstrated in *P. patens* that episomal recombination events can occur in a reproducible and predictable manner, guided by as few as a 12 bases of homology^18^. We therefore investigated whether the homologous recombination machinery of *P. patens* can be utilized to assemble and integrate DNA fragments with short overlapping sequences. When the assembled multi-fragment construct contains 5′ and 3′ flanking sequences with homology to the *P. patens* genome, it integrates in the genome in a targeted manner, similar to the typical transformation with a linearized vector. The short length requirement for efficient homologous recombination is a significant technological advance, since short (12–20) base overlaps are convenient to incorporate into PCR primers. Complex *in vivo* assembly of multiple DNA fragments is a routine procedure using *S. cerevisiae*, contributing greatly to its extensive use as a synthetic biology and biotechnology host^19^,^20^,^21^, therefore the development of a similar methodology for *P. patens* can further increase its utility as a plant biotechnological chassis. Here, we explore direct PEG transformation of *P. patens* using PCR products containing short overlapping sequences, and show that it is a robust method for genome engineering.

## Results and Discussion

**Figure 1:**
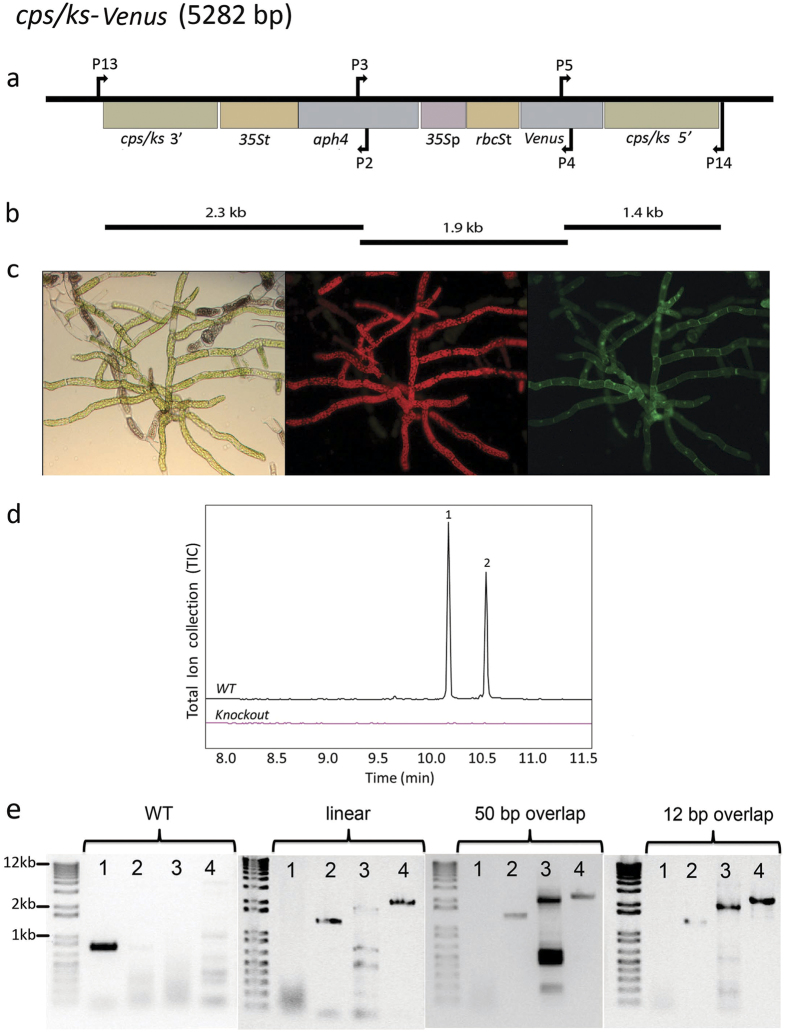
Knockout of the native diterpene synthase *CPS/KS* gene. **(a)** A vector map of the linearized pBK3 vector, including 1000 bp both 5′ and 3′ of the native *CPS/KS* coding sequence. The 5′ region contains the 1491 bp of the region upstream the ATG for this diterpene synthase gene fused to the gene for the fluorescent protein, Venus. Resistance to hygromycin was conferred by aminoglycoside phosphotransferase 4 coded by *aph4* under expression from the *35S* promoter. Arrows indicate locations of primer pairs used for PCR genotyping. All genetic maps were created using the software OG Draw (http://ogdraw.mpimp-golm.mpg.de/). **(b)** Depiction of the three PCR fragments amplified from pBK3, of sizes 2.3, 1.9, and 1.4 kb. **(c)** Microscopy of *Ppcps/ks* knockout plants. From left to right: Bright-field, chlorophyll fluorescence (590–680 nm) and Venus fluoresence (520–560 nm). **(d)** GC-MS analysis of WT *P. patens* and a multi-fragment transformation resulting in the absence of the two major diterpenes found in hexane extractions, 16-*ent*-kaurene (1) and 16-OH-*ent*-kaurene (2). **(e)** Proper insertion and *in vivo* assembly of DNA fragments were confirmed by PCR. Primer pair 1 (P13 andP14) only binds in a region of DNA specific to the wild type (WT) *CPS/KS* locus, and gives a band of 778 bp only if the WT locus is intact. This band is absent in the three shown transformed lines, including a linearized plasmid transformant, a 50 bp overlap transformant, and a 12 bp overlap transformant. The primer pairs 2 (P14, P5), 3 (P3, P4) and 4 (P13, P2) give sizes of 1421 bp, 1882 bp, and 2372 bp respectively, indicating proper assembly of fragments and insertion in the genome. The PCR products were sequence-verified.

To demonstrate uptake, assembly, and correct genomic integration of multiple DNA fragments in moss, we focused on three examples of *P. patens* transformation related to plant biotechnology. We utilized our published vector pBK3 that was established in order to study terpenoid biosynthesis through the knock-out of the native *P. patens* bifunctional copalyl diphosphate/kaurene synthase (*CPS/KS*) gene^7^,^22^,^23^. The vector replaces *CPS/KS* with the gene for the enhanced yellow fluorescent protein variant SEYFP-F46L (Venus)^24^ under hygromycin selection (Fig. 1a). Using this vector as a template, we amplified three DNA sequences (Fig. 1b) that cover the whole insert region, each having a sequence overlap with the one next to it. Four different overhang lengths (12, 20, 50, and 100 base pairs) were examined.

A correct transformation event results in stable *P. patens* lines that have three distinct phenotypes. They are resistant to hygromycin, express *Venus* from the *CPS/KS* promoter, and lack *ent*-kaurene derived diterpenes (Fig. 1c,d). We transformed *P. patens* with linear pBK3 vector and with equimolar amounts of the PCR fragments produced from the same vector. All the PCR fragments were also transformed individually as negative controls (Supplemental Fig. 1). As the coding sequences of both the Venus reporter and the hygromycin resistance are split on different DNA fragments, and none of the fragments contain a full-length coding sequence, the DNA repair system of *P. patens* must assemble the three fragments via homologous recombination to gain all the phenotypes described above. We recovered 12–21 (Table 1) antibiotic resistant lines for the different transformation approaches, the linear vector-transformation and the *in vivo* assembly (Fig. 1c). We then screened all of these resistant lines for fluorescent protein expression. Transformations using single fragments did not result in plants expressing *Venus* or surviving antibiotic selection. Accumulation of Venus was observed in both protonemal tissue (Fig. 1c) and gametophores (Supplemental Fig. 2). However, accumulation of Venus was only observed in rapidly dividing cells, such as protonemal tips and the core of the gametophore, tissue giving rise to the sporophyte. This suggests that the CPS/KS enzyme and *ent*-kaurene related metabolites are associated with cell division and differentiation, providing further insights in the functionality of *ent*-kaurenes in *P. patens* and other bryophytes. We performed metabolite profiling of the fluorescent lines, and confirmed the lack of detectable diterpenoids in 6–11 lines using the different transformation approaches (Table 1, Fig. 1d). In order to verify the correct insertion of the transgenes, we genotyped the lines that were both fluorescent and lacking *ent*-kaurenes, (Fig. 1e). Sequence information was obtained for the regions where the DNA assembly occurred. Two fully genotyped lines were recovered showing the appropriate phenotypes with linear vector-transformation and 4 lines of the different *in vivo* assembly approaches, the fragments were correctly assembled and integrated in the targeted genetic locus, and lacked the native *CPS/KS* gene. The correctly integrated and assembled transformants (for both linear vector and multi-fragment transformation) correspond to 10–22% of the antibiotic resistant lines. The transformation results and the number of obtained lines are summarized in Table 1.

**Figure 2:**
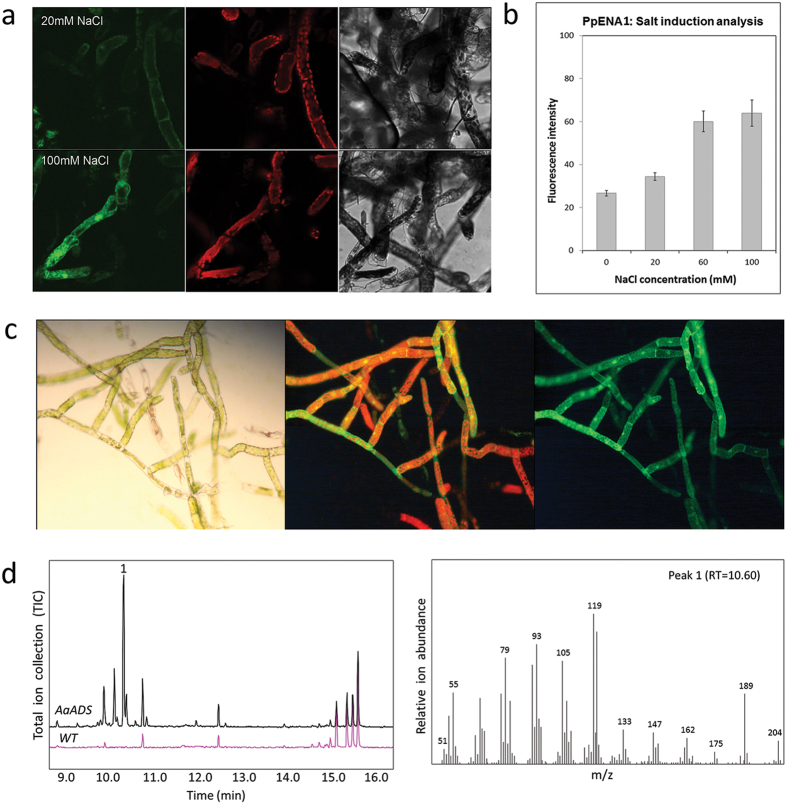
Transformation of *P. patens* with the endogenous salt-inducible promoter, *ena1*, and the amorphadiene synthase gene *ADS* from *Artemisia annua*, targeted to the neutral site Pp108. (**a**) Venus fluorescence after 24 h induction of *PpENA1*-*Venus* expression with 20 mM, 60 mM, and 100 mM NaCl (0 mM was used as negative control) was observed in both protonemal tissue and gametophores. Three fluorescent wavelengths were tested (from left to right): Bright field, chlorophyll fluorescence (590–680 nm), and Venus fluorescence (520–560 nm). (**b**) Graph showing the fluorescent intensity of Venus, after the expression of its gene was induced by different salt concentrations. (**c**) Fluorescence of AaADS-Venus. From left to right: UV light (350–420 nm), chlorophyll fluorescence (590–680 nm) and Venus fluorescence (Venus) (520–560 nm). (**d**) GC-MS analysis of amorphadiene (1) produced from transgenic *P. patens* with mass spectra and retention time.

We also generated two novel constructs that were designed for integration in the well-established neutral locus Pp108 that allows efficient genomic integration in *P. patens*^3^. Here, we amplified the overlapping DNA fragments, adding 27–30 base overhangs via PCR primers for transformation without pre-assembly of vectors in *E. coli*. As inducible systems are valuable for tuning of heterologous expression, we studied the *P. patens* sodium inducible promoter, *PpENA1*^25^ with *Venus* as a reporter gene. For selection, this three-fragment assembly encoded a geneticin (G418) resistance (*nptII*) cassette (Supplemental Fig. 3). We obtained 32 fluorescent lines, of which six were assembled correctly. The promoter activity was measured in plants growing on media supplemented with NaCl (0–100 mM). We observed NaCl induced *Venus* expression (Fig. 2a), which reached a plateau at a concentration of 60 mM NaCl (Fig. 2b). These results are consistent with previously reported data on this promoter^25^.

Finally, we expressed the gene for the amorphadiene synthase AaADS, a sesquiterpene synthase from *Artemisia annua* that catalyzes the first committed step of the biosynthesis of the antimalarial drug artemisinin^26^. AaADS was expressed coupled to Venus using the LP4 linker (a linker peptide from a polyprotein precursor of *Impatiens balsamina*, which results in expression of two separate proteins^27^,^28^). The promoter driving the fused genes was the *UBIQUITIN1* promoter from *Zea mays* (*ZmUBI1*), and the selection was again based on geneticin resistance. Collectively, these PCR products gave a four-fragment assembly (Supplemental Fig. 4). We analyzed the 10 lines showing antibiotic resistance for yellow fluorescence (Fig. 2c), and we subjected the resulting 9 fluorescent lines to GC-MS analysis to confirm biosynthesis of amorphadiene by the AaADS (Fig. 2d). Correct insertions in the Pp108 locus and assembly of both the *PpENA1* and the *AaADS* constructs were confirmed for three independent lines via genotyping. Assembly and integration were tested by PCR-based genotyping and sequence-verification (Supplemental Figs 5 and 6). In these cases, assembly was demonstrated by appropriate phenotypes and PCR confirmation of internal junctions, however genomic integration could not be verified. This suggests the possibility that assembled DNA was maintained episomally, without integration to the genome.

In all three transformation experiments, we acquired plant lines that contain the desired genomic sequence, assembled correctly and inserted in the targeted genomic locus (Table 1). The evaluation of the transformant phenotypes was performed sequentially, i.e. resistant lines were screened for fluorescence, followed by metabolite profiling using GC-MS, before genotyping. The transformation efficiency and correct integration were similar for the multi-fragment transformations and the traditional transformation using a linearized vector. Previous studies indicated that the specific targeted locus and the presence of repeating elements are possibly more important determinants of the transformation efficiency than the length of the homology regions^17^,^29^.

Previous studies also reported incomplete and non-targeted integration. Episomal concatamers and tandem insertions are common events, and were suggested as result from non-homologous end-joining. In addition, one-sided integration and random insertions were described^18^,^29^,^30^. Consistent with such non-targeted and incomplete integration events, we observed antibiotic resistant plant lines lacking one or more of the independent phenotypes used (Table 1). However, the three examples of transformation presented here demonstrate the ability of *P. patens* to take up, assemble and correctly integrate up to 9.2 kilobases of DNA directly into the genomic target site. This new transformation technology provides an easy way to assemble DNA fragments in a plant system, minimizing the need for laborious sub-cloning and gene construction in *E. coli* or *S. cerevisiae* vectors. Moreover, using this technique allows for flexible genome editing, combinatorial assembly of DNA parts, and engineering of complex metabolic pathways, which are all important for high-throughput synthetic biology studies.

Improved techniques for targeted, controlled, and large-scale genome manipulation of plant systems are highly valuable and could facilitate understanding and improving genetic structure and function at the chromosome and genome levels. *P. patens* is already extensively used for both basic research and applied biotechnology. The implementation and optimization of *in vivo* DNA assembly methodologies can significantly improve the potential of *P. patens* as a biotechnological host species and as a photosynthetic chassis for synthetic biology.

## Methods

### Plant material

*P*. *patens* (Gransden ecotype, International Moss Stock Center #40001) was grown on solid PhyB media^14^,^15^, in a growth chamber, at 25 °C with 16/8 h light/dark cycle, 20–50 W/m^2^ light intensity.

### Transformation of *P. patens* protoplasts

Protoplasts were prepared and transformed using a standard PEG method^14^,^15^. More precisely, approximately 1.5 g in fresh weight (three Petri dishes) of 5 days cultured *P*. *patens* was digested with a 0.5% Driselase® enzyme solution in 8.5% mannitol (Sigma D9515), using one ml of solution for every 40 mg *P. patens* tissue and incubated with gentle agitation for 30–60 minutes at room temperature. The digested tissue was filtered through mesh with pore size of 100 μm. Protoplasts were pelleted by centrifuging at 150–200 × g for 5 minutes with slow breaking. The supernatant was discarded and the pellet was washed twice with protoplast wash solution (8.5% mannitol, 10 mM CaCl_2_). Protoplast density was measured using a hemocytometer, followed by centrifuging and resuspension in MMM solution (9.1% D-mannitol, 10% MES and 15mM MgCl_2_) giving a protoplast concentration of 1.6 × 10^6^ cells/mL. 300 μL of protoplast suspension and 300 μL of PEG solution were added to a 15 mL tube containing the DNA. For both linearized plasmid and multi-fragment transformations, 20 μg total DNA was used. For the multi-fragment transformations, fragments were added in an equimolar ratio.

The mixture was incubated in a 45 °C water bath for 5 minutes and another 5 minutes at room temperature. Samples were diluted 5 times by adding 300 μL of 8.5% D-mannitol with 1 min pauses between dilutions, followed by an additional 5 dilutions with 1 mL of 8.5% D-mannitol. The transformed protoplasts were pelleted by centrifugation and the supernatant was discarded. The protoplast pellet was resuspended in 500 μL of 8.5% D-mannitol and 2.5 mL of protoplast regeneration media (top layer; PRMT). 1 ml of the suspension was dispensed on each plate (three plates in total) containing protoplast regeneration media (bottom layer; PRMB) overlaid with a cellophane disc. The plates were incubated for 5–7 days. The cellophane with regenerating protoplasts was then transferred on PhyB media containing appropriate selection for two weeks.

Transformations were moved to media without antibiotics for another two weeks to allow loss of unstable and non-integrated DNA, and then plants were moved back to a final antibiotic selection for two weeks at which point they were considered stable. A detailed protocol can be found in references^14^,^15^.

### DNA parts and genes

Genomic sequences and annotations of *P. patens* were retrieved form cosmoss.org. The pBK3 vector^7^ was used to generate the *Ppcps/ks*-knockout plants. The *PpENA1* promoter and the homologous recombination flanking regions of the Pp108 locus were amplified from *P. patens* genomic DNA. The *AaADS* template was a kind gift from Assoc. Prof. Dae Kyun Ro, University of Calgary, Canada. The *ZmUBI1* promoter was obtained from the pMP1355 vector, a kind gift from Professor Mark Estelle, University of California San Diego, USA.

### PCR, DNA purification and concentration

Polymerase chain reactions (PCR) were performed using Phusion® High-Fidelity DNA Polymerase (New England Biolabs) or Hotmaster Taq (5PRIME) according to provider’s protocol. The primers used are listed in Supplemental Table 1. PCR products for transformations or sequencing were purified using the QIAquick PCR Purification Kit (Qiagen), according to manufacturer’s protocol. Purified DNA for transformations was vacuum-concentrated in a ScanVac vacuum concentrator (LaboGene) to a final DNA concentration of ~1 μg/μL determined spectrophotometrically using NanoDrop2000 (Thermo Fisher Scientific).

### Microscopy

Transformed *P. patens* lines were screened using a stereo microscope (Leica MZ FLII Stereo Fluorescence Microscope) and a fluorescence source (Leica DN 5000B Fluorescence Microscope). The *Ppcps/ks* knockout lines were sub-cultured on solid PhyB media while the *PpENA1* lines were sub-cultured on media containing NaCl (0 mM (non induced), 20 mM, 60 mM, and 100 mM) with wild-type as a control. Micrographs were captured 24 hours after induction. Fluorescence intensity of approximately 25 replicates from each treatment was quantified with the software program Fiji (ImageJ).

The *Ppcps/ks* knockout and *PpENA1-Venus* lines were further analyzed with a Leica TCS SP5 II spectral confocal laser-scanning microscope (Leica Microsystems, Heidelberg, Germany). The fluorescence was quantified with a 63x/1.2 numerical aperture water immersion objective for the detection of yellow fluorescence. A sequential scanning was performed for the *PpENA1* lines, to detect the fluorescence of the Venus protein, whose expression was driven by the inducible *PpENA1* promoter. The excitation was set at 488 nm allowing for the detection of chlorophyll at 590–680 nm. This was followed by excitation 515 nm that allow for the detection of Venus at 520–560 nm. The maximum intensity was set as the relative fluorescence exhibited from cells treated with 100 mM NaCl.

### Metabolite profiling

The metabolite profile of the transgenic lines was examined using a Shimadzu GCMS-QP2010 Plus (GC-2010). Samples were prepared as previously described^14^. To screen for biochemical knockouts, one microliter of hexane extract was injected in split-less mode and separated with HP-5MS UI column (20.0 m × 0.18 mm × 0.18 μm) with hydrogen as a carrier gas. The GC program was as follows: 60 °C for 3 min, 60~150 °C at 30 °C/min, 150~210 °C at 10 °C/min, 210~320 °C at 30 °C/min and was held for 5 min. The profile of *AaADS* lines was determined by HS-SPME (Headspace-Solid Phase Micro-Extraction)^31^,^32^. The identity of amorphadiene and *ent*-kaurene was confirmed by comparison of the obtained mass spectrum and retention index with those found in the literature^33^ and the NIST 08 and Wiley 8.0 libaries.

### DNA isolation

DNA for PCR analysis was isolated as previously described^34^, with minor modifications. Briefly, fresh *P. patens* tissue (approximately 10–20 mg) was collected in 1.5 ml microfuge tubes, frozen in liquid nitrogen, then ground using a plastic pestle. 400 μL of extraction buffer (50 mM Tris·HCl pH 8.0 and 20 mM EDTA pH 8.0) was added followed by 80 μl of 10% SDS. The mixture was gently vortexed and incubated at 65 °C for 30 min. 180 μL of 3M NaAc pH 5.2 was added to the mixture and samples were incubated on ice for 20 min. Samples were centrifuged at 15,000 × *g* for 10 min to pellet debris. The supernatant was transferred to a new tube containing an equal volume of isopropanol and was mixed by inversion. DNA was pelleted by centrifugation at 15,000 × *g* for 30 min. The supernatant was discarded and the pellet was washed with 80% ethanol and allowed to air dry before the final resuspension in 50 μL water.

## Additional Information

**How to cite this article**: King, B. C. *et al. In vivo* assembly of DNA-fragments in the moss, *Physcomitrella patens*. *Sci. Rep.* **6**, 25030; doi: 10.1038/srep25030 (2016).

## Supplementary Material

Supplementary Information

## Figures and Tables

**Table 1:** Number of *P. patens* lines generated in this work and comparison of transformation efficiency.

	Resistant lines	Fluorescent lines	GC-MS positive lines	Genotype-positive lines^a^	Efficacy [%]^b^
linearized pBK3	20	14	6	2	10.0
*In vivo* assembly pBK3 (12 bp overlap)	25	21	11	4	16.0
*In vivo* assembly pBK3 (20 bp overlap)	18	14	6	4	22.2
*In vivo* assembly pBK3 (50 bp overlap)	27	19	9	4	14.8
*In vivo* assembly pBK3 (100 bp overlap)	19	12	8	4	21.1
*PpENA1*-*Venus*	32	32	c	6	18.8
*AaADS*	10	9	7	3	30.0

^a^Plant lines, in which the native locus replacement and proper assembly were confirmed.

^b^Defined as genotype-positive/resistant lines.

^c^No product was expected for the *PpENA-Venus* transformants.

## References

[b1] Reski R., Parsons J. & Decker E. L. Moss-made pharmaceuticals: from bench to bedside. Plant Biotechnology Journal 13, 1191–1198 (2015). 26011014 10.1111/pbi.12401 PMC4736463

[b2] Rensing S. A. *et al.* The *Physcomitrella* genome reveals evolutionary insights into the conquest of land by plants. Science 319, 64–69 (2008). 18079367 10.1126/science.1150646

[b3] Schaefer D. G. & Zrÿd J. P. Efficient gene targeting in the moss *Physcomitrella patens*. The Plant journal 11, 1195–1206 (1997). 9225463 10.1046/j.1365-313x.1997.11061195.x

[b4] Lang D., Zimmer A. D., Rensing S. A. & Reski R. Exploring plant biodiversity: the *Physcomitrella* genome and beyond. Trends in Plant Science 13, 542–549 (2008). 18762443 10.1016/j.tplants.2008.07.002

[b5] Vesty E. F. *et al.* The decision to germinate is regulated by divergent molecular networks in spores and seeds. New Phytologist in press (2016). 10.1111/nph.14018 PMC4950004 27257104

[b6] Decker E. L., Parsons J. & Reski R. Glyco-engineering for biopharmaceutical production in moss bioreactors. Front Plant Sci 5, 346 (2014). 25071817 10.3389/fpls.2014.00346 PMC4089626

[b7] Pan X.-W., Han L., Zhang Y.-H., Chen D.-F. & Simonsen H. Sclareol production in the moss *Physcomitrella patens* and observations on growth and terpenoid biosynthesis. Plant Biotechnol Rep 9, 1–11 (2015).

[b8] Zhan X., Han L. A., Zhang Y., Chen D. & Simonsen H. T. Metabolic engineering of the moss *Physcomitrella patens* to produce the sesquiterpenoids patchoulol and α/β‐santalene. *Front* Plant Sci 5, 636 (2014). 10.3389/fpls.2014.00636 PMC4235272 25477891

[b9] Anterola A., Shanle E., Perroud P.-F. & Quatrano R. Production of taxa-4(5),11(12)-diene by transgenic *Physcomitrella patens*. Transgenic Res 18, 655–660 (2009). 19241134 10.1007/s11248-009-9252-5

[b10] Decker E. L. & Reski R. The moss bioreactor. Current Opinion in Plant Biology 7, 166–170 (2004). 15003217 10.1016/j.pbi.2004.01.002

[b11] Lassen L. M. *et al.* Redirecting Photosynthetic Electron Flow into Light-Driven Synthesis of Alternative Products Including High-Value Bioactive Natural Compounds. ACS Synthetic Biology 3, 1–12 (2014). 24328185 10.1021/sb400136f

[b12] Ikram N. K. B. K., Zhan X., Pan X., King B. C. & Simonsen H. T. Stable Heterologous Expression of Biologically Active Terpenoids in Green Plant Cells. Front Plant Sci 6 (2015). 10.3389/fpls.2015.00129 PMC4364152 25852702

[b13] Simonsen H. T., Drew D. P. & Lunde C. Perspectives on Using *Physcomitrella Patens* as an Alternative Production Platform for Thapsigargin and Other Terpenoid Drug Candidates. Perspectives in Medicinal Chemistry 3, 1–6 (2009). 19812738 10.4137/pmc.s2220 PMC2754923

[b14] Bach S. S., King B. C., Zhan X., Simonsen H. T. & Hamberger B. In Plant Isoprenoids Vol. 1153 (ed. Rodríguez-Concepción M. ) 257–271 (Springer New York, New York, USA; 2014).

[b15] Cove D. J. *et al.* Transformation of the Moss *Physcomitrella patens* Using Direct DNA Uptake by Protoplasts. Cold Spring Harbor Protocols 2009, pdb.prot5143 (2009). 10.1101/pdb.prot5143 20147073

[b16] Hohe A. *et al.* An improved and highly standardised transformation procedure allows efficient production of single and multiple targeted gene-knockouts in a moss, Physcomitrella patens. Current Genetics 44, 339–347 (2004). 14586556 10.1007/s00294-003-0458-4

[b17] Schaefer D. G. Gene targeting in *Physcomitrella patens*. Current Opinion in Plant Biology 4, 143–150 (2001). 11228437 10.1016/s1369-5266(00)00150-3

[b18] Murén E., Nilsson A., Ulfstedt M., Johansson M. & Ronne H. Rescue and characterization of episomally replicating DNA from the moss *Physcomitrella*. Proceedings of the National Academy of Sciences 106, 19444–19449 (2009). 10.1073/pnas.0908037106 PMC2780769 19892729

[b19] Shao Z., Zhao H. & Zhao H. DNA assembler, an *in vivo* genetic method for rapid construction of biochemical pathways. Nucleic Acids Research 37, e16 (2009). 19074487 10.1093/nar/gkn991 PMC2632897

[b20] Gibson D. G. *et al.* Complete chemical synthesis, assembly, and cloning of a *Mycoplasma genitalium* genome. Science 319, 1215–1220 (2008). 18218864 10.1126/science.1151721

[b21] Annaluru N. *et al.* Total synthesis of a functional designer eukaryotic chromosome. Science 344, 55–58 (2014). 24674868 10.1126/science.1249252 PMC4033833

[b22] Zhan X., Bach S. S., Hansen N. L., Lunde C. & Simonsen H. T. Additional diterpenes from *Physcomitrella patens* synthesized by copalyl diphosphate/kaurene synthase (PpCPS/KS). Plant Physiology and Biochemistry 96, 110–114 (2015). 26248039 10.1016/j.plaphy.2015.07.011

[b23] Hayashi K. *et al.* Identification and functional analysis of bifunctional ent-kaurene synthase from the moss *Physcomitrella patens*. FEBS Letters 580, 6175–6181 (2006). 17064690 10.1016/j.febslet.2006.10.018

[b24] Nagai T. *et al.* A variant of yellow fluorescent protein with fast and efficient maturation for cell-biological applications. Nature Biotechnology 20, 87–90 (2002). 10.1038/nbt0102-87 11753368

[b25] Lunde C., Drew D. P., Jacobs A. K. & Tester M. Exclusion of Na^+^ via sodium ATPase (PpENA1) ensures normal growth of *Physcomitrella patens* under moderate salt stress. Plant Physiology 144, 1786–1796 (2007). 17556514 10.1104/pp.106.094946 PMC1949878

[b26] Wallaart T. E., Bouwmeester H. J., Hille J., Poppinga L. & Maijers N. C. Amorpha-4,11-diene synthase: cloning and functional expression of a key enzyme in the biosynthetic pathway of the novel antimalarial drug artemisinin. Planta 212, 460–465 (2001). 11289612 10.1007/s004250000428

[b27] François I. E. J. A. *et al.* Processing in *Arabidopsis thaliana* of a heterologous polyprotein resulting in differential targeting of the individual plant defensins. Plant Science 166, 113–121 (2004).

[b28] Tailor R. H. *et al.* A novel family of small cysteine-rich antimicrobial peptides from seed of *Impatiens balsamina* is derived from a single precursor protein. J Biol Chem 272, 24480–24487 (1997). 9305910 10.1074/jbc.272.39.24480

[b29] Kamisugi Y. *et al.* The mechanism of gene targeting in *Physcomitrella patens*: homologous recombination, concatenation and multiple integration. Nucleic Acids Research 34, 6205–6214 (2006). 17090599 10.1093/nar/gkl832 PMC1693892

[b30] Wendeler E., Zobell O., Chrost B. & Reiss B. Recombination products suggest the frequent occurrence of aberrant gene replacement in the moss *Physcomitrella patens*. Plant Journal 81, 548–558 (2015). 25557140 10.1111/tpj.12749

[b31] Drew D. P., Rasmussen S. K., Avato P. & Simonsen H. T. A comparison of headspace solid-phase microextraction and classic hydrodistillation for the identification of volatile constituents from *Thapsia* spp. provides insights into guaianolide biosynthesis in Apiaceae. Phytochemical Analysis 23, 44–51 (2012). 21618308 10.1002/pca.1323

[b32] Andersen T. B., Cozzi F. & Simonsen H. T. Optimization of biochemical screening methods for volatile and unstable sesquiterpenoids using HS-SPME-GC-MS. Chromatography 2, 277–292 (2015).

[b33] Adams R. P. Identification of Essential Oil Components by Gas Chromatography/Mass Spectrometry, Edn. 4th (Allured Pub Corp, Carol Stream, IL, USA; 2007).

[b34] Dellaporta S., Wood J. & Hicks J. A plant DNA minipreparation: Version II. Plant Mol Biol Rep 1, 19–21 (1983).

