# Corrigendum: *In vivo* assembly of DNA-fragments in the moss, *Physcomitrella patens*

**DOI:** 10.1038/srep31261

**Published:** 2016-08-25

**Authors:** Brian Christopher King, Konstantinos Vavitsas, Nur Kusaira Binti Khairul Ikram, Josephine Schrøder, Lars B. Scharff, Jean-Étienne Bassard, Björn Hamberger, Poul Erik Jensen, Henrik Toft Simonsen

Scientific Reports 6: Article number: 25030; 10.1038/srep25030published online: 04 29 2016; updated: 08 25 2016

Jean-Étienne Bassard was omitted from the author list in the original version of this Article. This has been corrected in the PDF and HTML versions of the Article.

The Acknowledgements section now reads:

Brian C. King and Henrik Toft Simonsen were supported by SpotLight, a grant from the Danish Council for Strategic Research. Brian C. King, Konstantinos Vavitsas, Jean-Étienne Bassard and Björn Hamberger were supported by the VILLUM research center of Excellence “Plant Plasticity”, the UCPH Excellence Programme for Interdisciplinary Research “bioSYNergy”, and by the Innovation Fund Denmark (previously the Danish Council for Strategic Research) grant “Plant Power: Light-Driven Synthesis of Complex Terpenoids Using Cytochromes P450” (12-131834). Nur Kusaira Binti Khairul Ikram was supported by a grant from Ministry of Higher Education, Malaysia and University of Malaya. Imaging data were collected at the Center for Advanced Bioimaging Denmark, University of Copenhagen. The authors would like to thank Drs. Mark Estelle and Dae-Kyun Ro for the ZmUBI1 and AaADS templates, respectively.

The Author Contributions section now reads:

B.C.K., K.V. and N.K.B.K.I. planned and performed the experiments, analyzed the data, and wrote the manuscript. J.S., L.B.S. and J-E.A.B. performed the experiments, analyzed the data, and reviewed the manuscript. B.H., P.E.J. and H.T.S. planned the experiments, analyzed the data, and reviewed the manuscript.

