# Supplementary material for: In vivo assembly of DNA-fragments in the moss, Physcomitrella patens
Source: Sci Rep. 2016 Apr 29;6:25030. doi: 10.1038/srep25030 (PMC4850407; doi:10.1038/srep25030)
Supplement: Supplementary Information [file srep25030-s1.pdf]

In vivo assembly of DNA-fragments in the moss, *Physcomitrella patens*

Brian Christopher King†[1], Konstantinos Vavitsas†[1], Nur Kusaira Binti Khairul Ikram†[1,2,3], Josephine Schrøder[1], Lars B. Scharff[1], Björn Hamberger[1], Poul Erik Jensen[1], and Henrik Toft Simonsen[3]*

[1]Copenhagen Plant Science Centre, VILLUM Research Center for Plant Plasticity, Center for Synthetic Biology "bioSYNergy", Department of Plant and Environmental Sciences, University of Copenhagen, Thorvaldsensvej 40, DK-1871 Frederiksberg C, Denmark

[2]Institute of Biological Sciences, Faculty of Science, University of Malaya, Kuala Lumpur, Malaysia

[3]Department of Systems Biology, Technical University of Denmark, Søltofts Plads, 2800 Kgs. Lyngby, Denmark

†Equally contributed to the work

*Corresponding Author:

Henrik Toft Simonsen, Ph.D.

Department of Systems Biology, Technical University of Denmark, Søltofts Plads, 2800 Kgs. Lyngby, Denmark Corresponding Author's Email: hets@dtu.dk

Supplementary Data for King et al.

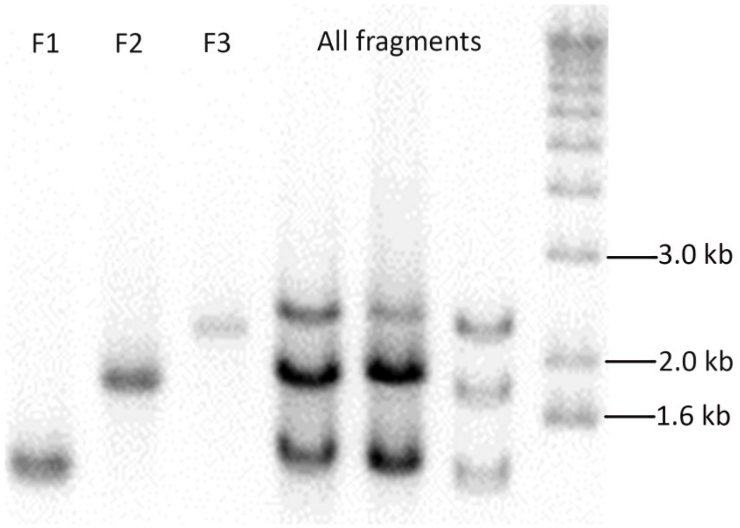

Supplementary Figure 1. Agarose gel showing DNA fragments to be used for the *cps/ks* knockout transformation experiments. Fragments 1-3 (F1-3), as described in Figure 1, were used to transform moss separately (negative controls) and all in combination in equimolar ratio.

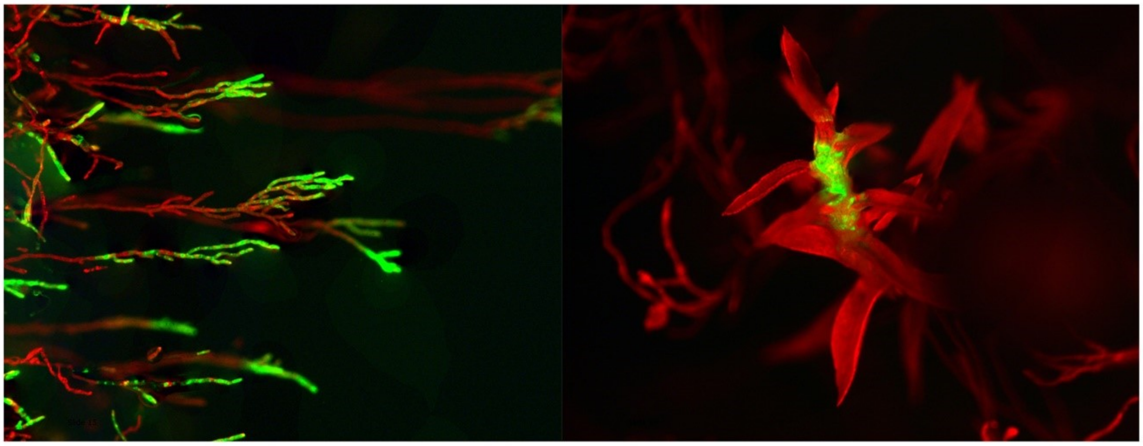

Supplementary Figure 2. Accumulation of Venus in both filamentous protonemal tissue and gametophores of a transformed moss line. Pictures captured with chlorophyll fluorescence (590-680 nm) and Venus fluorescence (Venus) (520-560 nm).

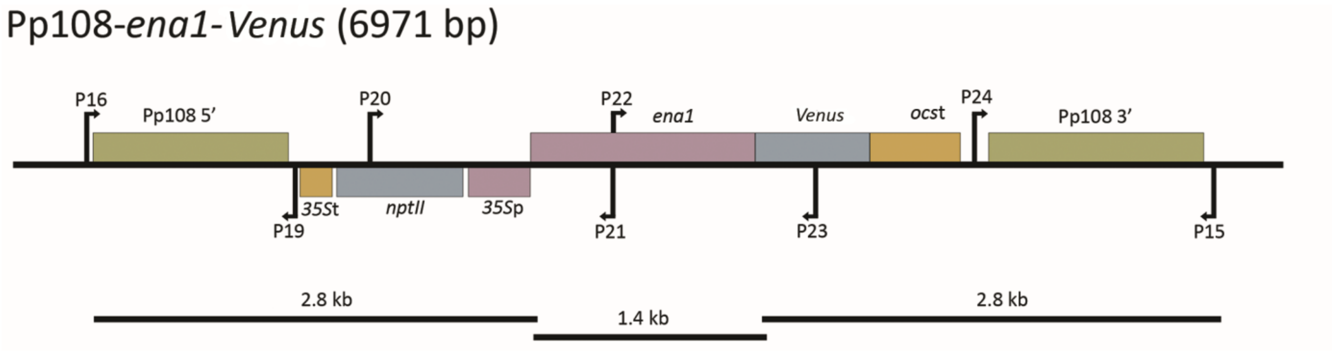

Supplementary Figure 3. Schematic representation of the *Ena1-Venus* DNA assembly in moss at the Pp108 neutral locus via three separate DNA fragments. Going from left to right, the first fragment contained the 5' Pp108 genome targeting sequence and the *nptII* resistance cassette, the second fragment contained the *ena1* promoter and the final fragment contained both *Venus* and the 3' Pp108 genome targeting sequence.

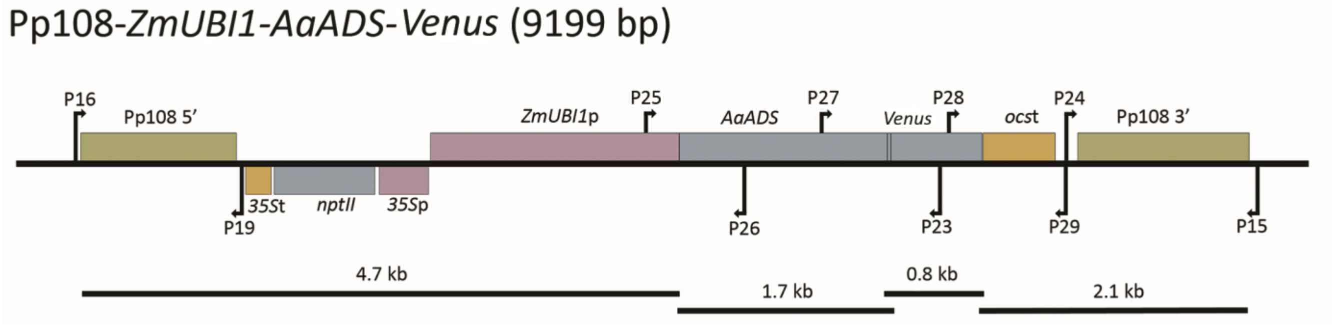

Supplementary Figure 4. Schematic representation of the *AaADS-Venus* DNA assembly in moss at the Pp108 neutral locus via four separate DNA fragments. Going from left to right, the first fragment contained the 5' Pp108 genome targeting sequence, the *35S* driven *nptII* resistance cassette and the *Zea mays UBIQUITIN I* promoter, the second fragment contained the *AaADS* gene and a sequence coding for the LP4 linker, the smaller 0.8 kb fragment contained the *Venus* coding region and the final fragment contained the *OCS* terminator and the 3' Pp108 genomic targeting sequence.

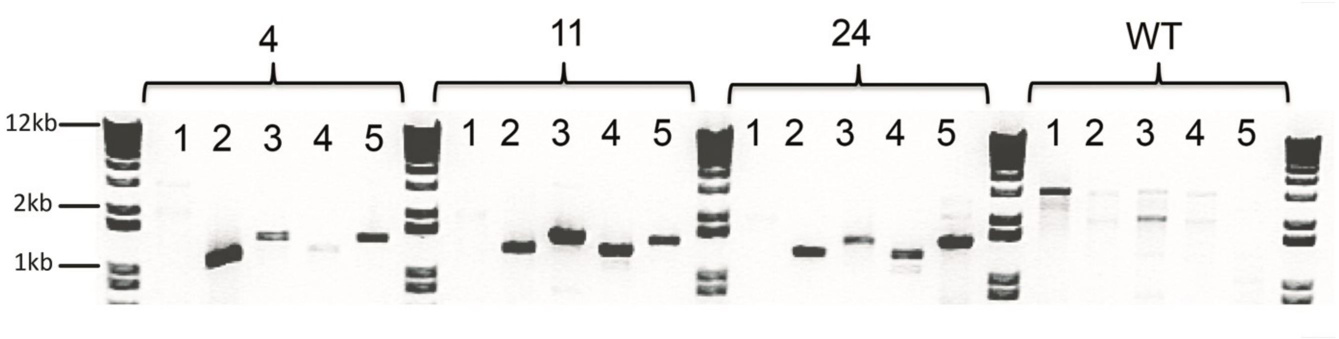

Supplementary Figure 5. *Ena1-Venus* insertion and assembly were confirmed by PCR followed by agarose gel electrophorsis. Primer pair of lane 1 (P15 and P16) only bind in a region of the genomic DNA specific to the wild type (WT) Pp108 locus, and give a band of 2580 bp only if the WT locus is intact. This band is absent in the three shown transformed lines. The primer pairs in lanes 2 (P16, P19), 3 (P20, P21), 4 (P22, P23) and 5 (P24, P15) (Supplemental Fig 3) give sizes of 1335, 1552, 1331 and 1525 bp respectively, indicating proper assembly of fragments and insertion in the genome.

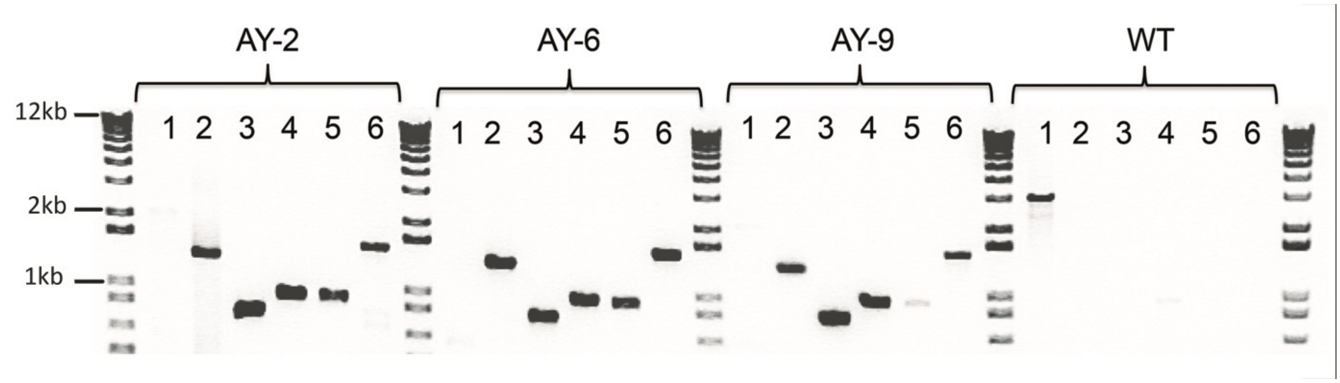

Supplementary Figure 6. *AaADS-Venus* DNA insertion and assembly were confirmed by PCR followed by agarose gel electrophorsis. Primer pair of lane 1 (P15 and P16) binds in a region of DNA specific to the wild type (WT) Pp108 locus, and give a band of 2580 bp only if the WT locus is intact. The primer pairs in lanes 2 (P16, P19), 3 (P25, P26), 4 (P27, P23), 5 (P28, P29) and 6 (P24, P15) (Supplemental Fig. 4) give sizes of 1335, 801, 949, 942 and 1525 bp, indicating proper assembly of fragments and insertion in the genome.

Primer Table

Name	Sequence	Comment
P1	CGAGAAGCATTAATGCGCAACCAC	Forward primer for pBK3 fragment 1
P2	CTCGATGCGGTTCACCAGGGTG	Reverse primer for pBK3 fragment 1
P3	AAGGCTACGTCCAGGAGCGCAC	Forward primer for pBK3 fragment 2 ,100bp overlap
P4	TCATCGAGAGCCTGCGCGACG	Reverse primer for pBK3 fragment 2
P5	GCGTGATTTCATATGCGCGATTGCTG	Forward primer for tpBK3 fragment 3,100bp overlap
P6	CATCCTTATTTCTCACTTTGTGCAACTTC	Reverse primer for pBK3 fragment 3
P7	GACCCGCGCCGAGGTGAAGTT	Forward primer for pBK3 fragment 2,50bp overlap
P8	ACTGTGATGGACGACACCGTCAG	Forward primer for pBK3 fragment 3,50bp overlap
P9	CACCCTGGTGAACCGCATCGAG	Forward primer for pBK3 fragment2,20bp overlap
P10	CGTCGCGCAGGCTCTCGATGA	Forward primer for pBK3 fragment 3,20bp overlap
P11	AACCGCATCGAGCTGAAGGGCAT	Forward primer for pBK3 fragment 2,12bp overlap
P12	GGCTCTCGATGAGCTGATGCTTTG	Forward primer for pBK3 fragment 3,12bp overlap
P13	TGCCTCCTGAAATAGTGGTTG	Genotyping primer, upstream of PpCPSKS locus
P14	CTACATGCTCAAAAACAAATGCTTG	Genotyping primer, downstream of PpCPSKS locus
P15	TGCTGCATTATGGATGGAAG	Genotyping primer, upstream of Pp108 locus
P16	CCAATCCGACCTCAAACAGAGC	Genotyping primer, downstream of Pp108 locus
P17	ACGTTTTCCAGGCACTAGAACTC	In PpCPSKS locus, to be removed by pBK3 insert
P18	CTTTAACACTCATGTAATCACATGTC	In PpCPSKS locus, to be removed by pBK3 insert
P19	CAATTCGCGGCCGATCTATAACTTC	In Pp108 locus, to be removed by insert
P20	CACCATGATATTCGGCAAGC	In G418 marker, downstream
P21	GGATGGTAGTAAGTGGGGGTGAAG	In PpENA1 promoter upstream
P22	CTTCACCCCCACTTACTACCATC	PpENA1 promoter downstream
P23	GAAGTCGATGCCCTTCAGCTCGATG	In YFP upstream
P24	CAGTGACGACAAATCGTTGG	Between OCS terminator and 108 locus 3', downstream
P25	CGATCTAGGATAGGTATACATGTTGATGTG	In ZmUbi downstream
P26	GTCATAATGGACAGACGAGAACGG	In ADS upstream
P27	CGTACGTAACCTGATGGTTGAAGC	In ADS downstream
P28	CACAACGTCTATATCACCGCCGACAAG	In Venus downstream
P29	CCAACGATTTGTCGTCACTGTCAAGG	Between OCS terminator and 108 locus 3', upstream
P30	GACCTGCAGAAGTAACACCAAACAACAG	ZmUBI reverse
P31	CCAGATCGGCCACATCCTTCTCCG	Pp108 5' primer forward
P32	ACGAAGGCCGTTCTTCCCTG	108_3' prime reverse
P33	GTCCTGCTTTAATGAGATATGCGAGACG	OCS terminator forward
P34	CCCTGTTGTTTGGTGTTACTTCTGCAGGTCATGGCCCTGACCGAAGAGAAAC	ZmUBI-ADS forward
P35	TGTAGCCACTTCATCTGCAGCATTAGAGATGGACATCGGGTAAACCAGCAG	ADS- LP4 fusion with Venus reverse primer
P36	TCTAATGCTGCAGATGAAGTGGCTACAATGGTGAGCAAGGGCGAGG	LP4-FP forward
P37	GGCGTCTCGCATATCTCATTAAAGCAGGACTTACTTGTACAGCTCGTCCATGCC	YFP-OCS fusion reverse
